# Supplementary material for: Impact on beer sales of removing the pint serving size: An A-B-A reversal trial in pubs, bars, and restaurants in England
Source: PLoS Med. 2024 Sep 17;21(9):e1004442. doi: 10.1371/journal.pmed.1004442 (PMC11407663; doi:10.1371/journal.pmed.1004442)
Supplement: S1 Appendix — (DOCX) [file pmed.1004442.s001.docx]

**Appendix S1 – Additional figures**

**Fig. A1 to A13­­:** Time series plots showing daily beer, wine and total sales, by site. Intervention period highlighted, along with occurrences of closure and special events. Protocol violation by site 2 noted.

**
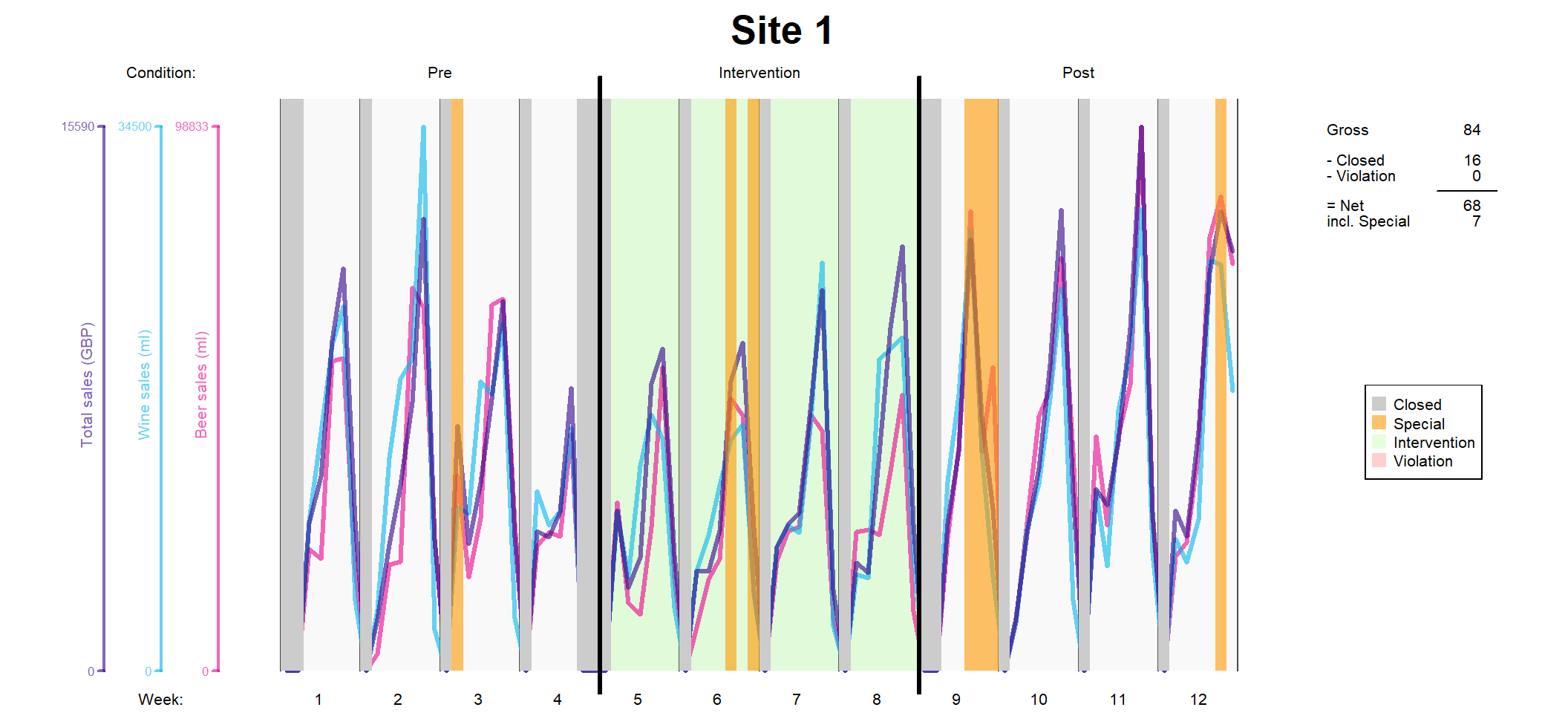

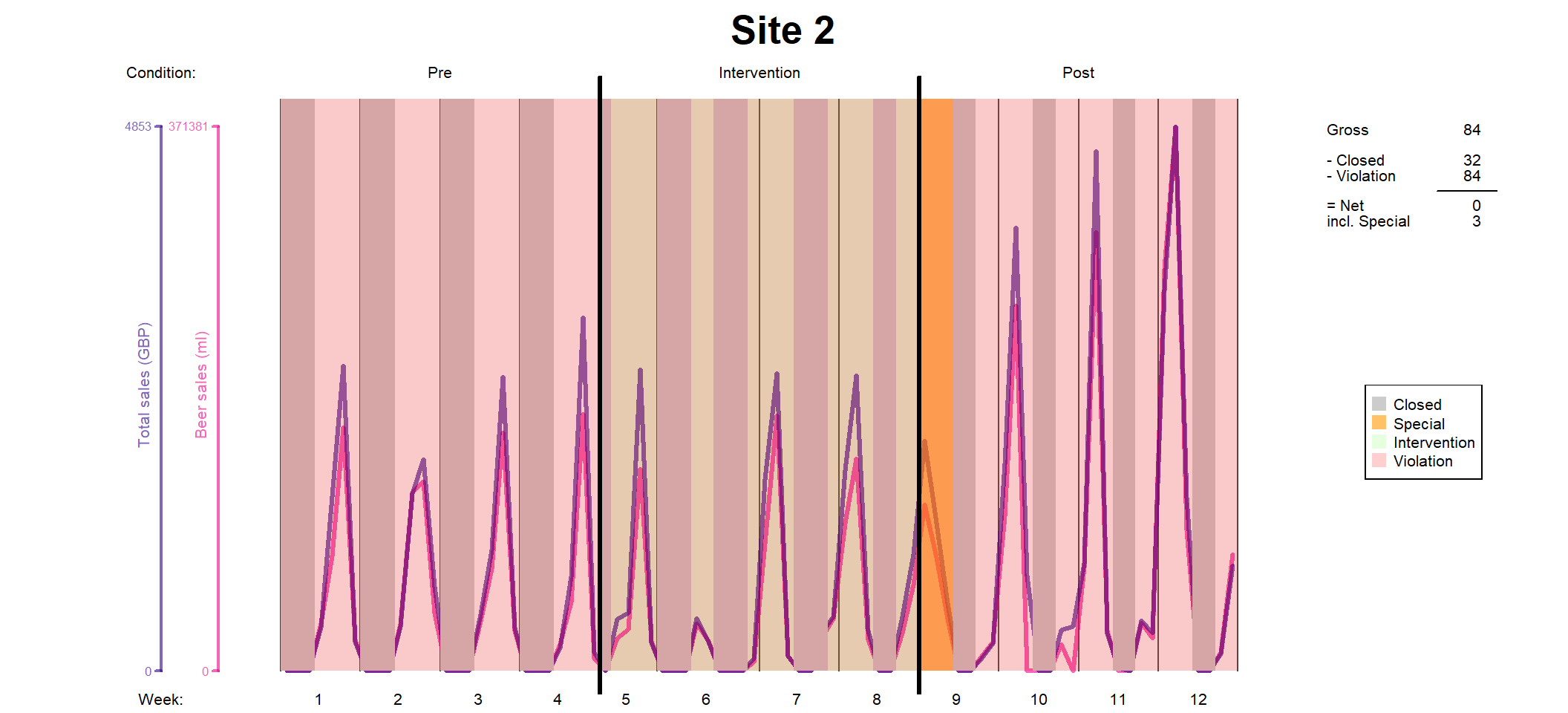

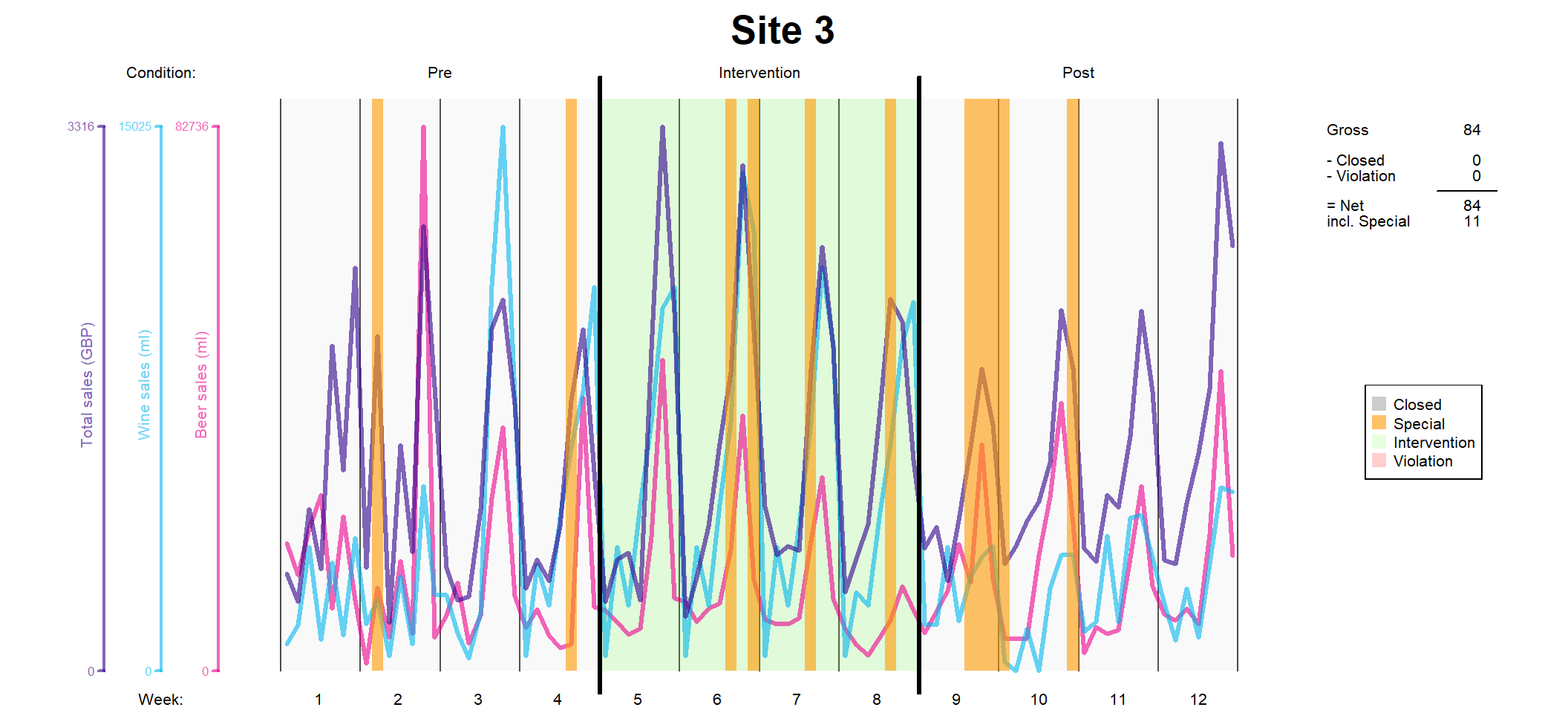

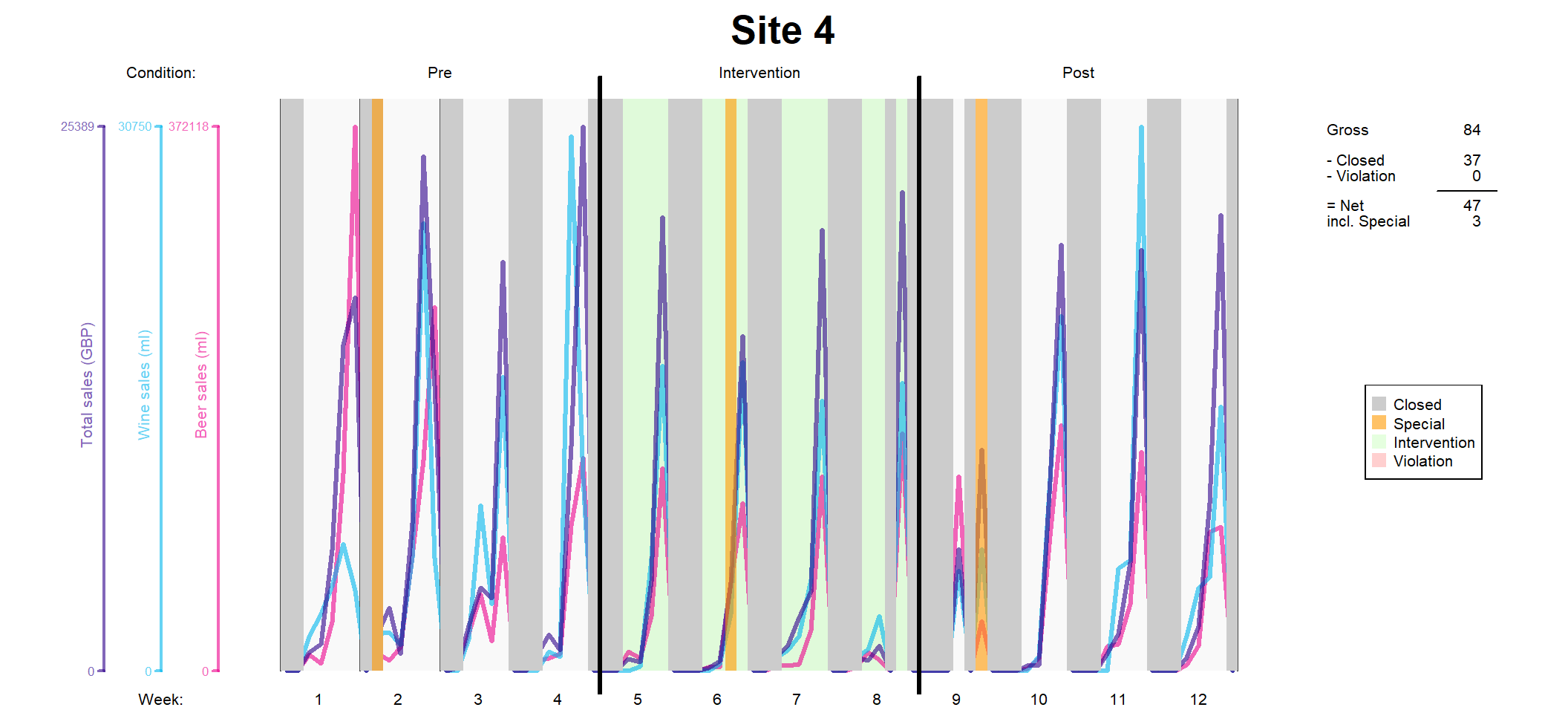

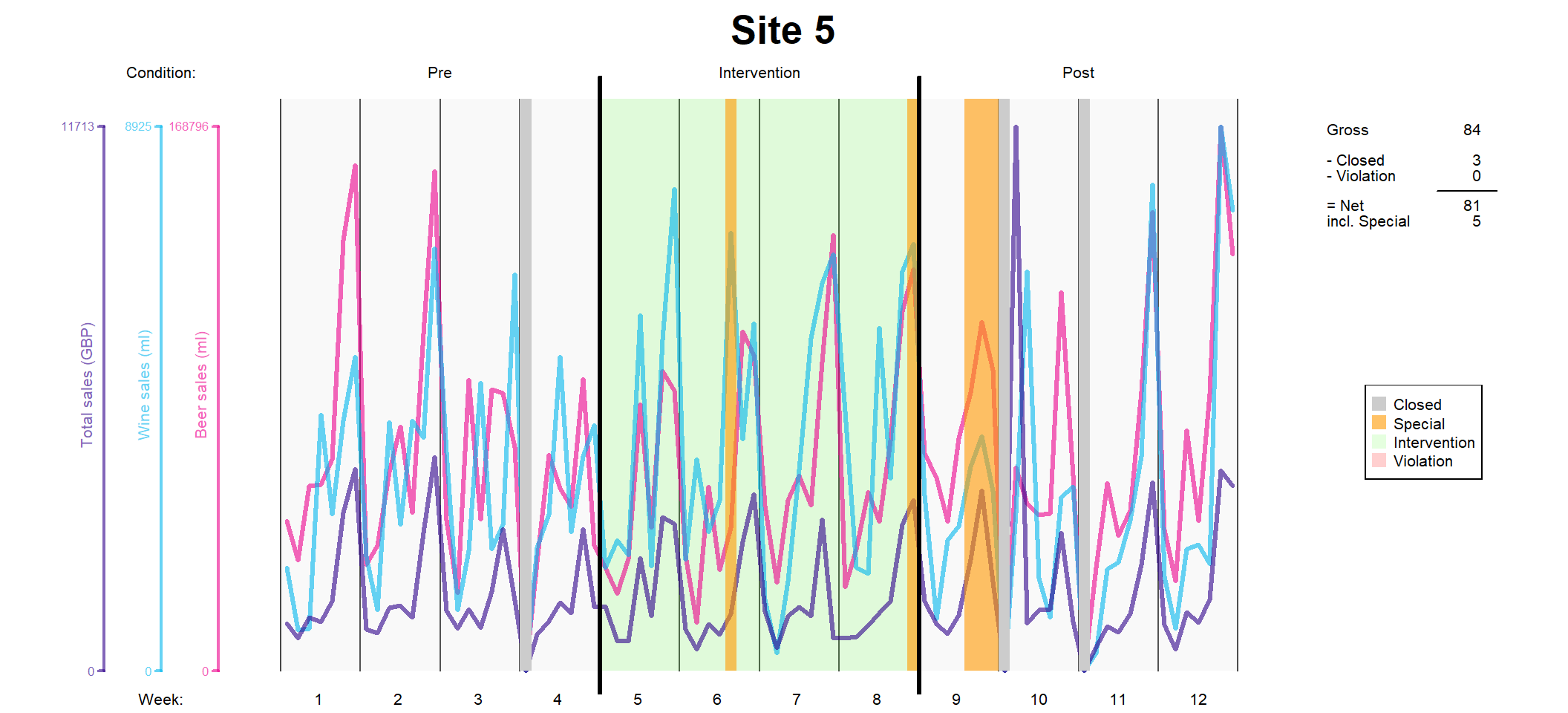

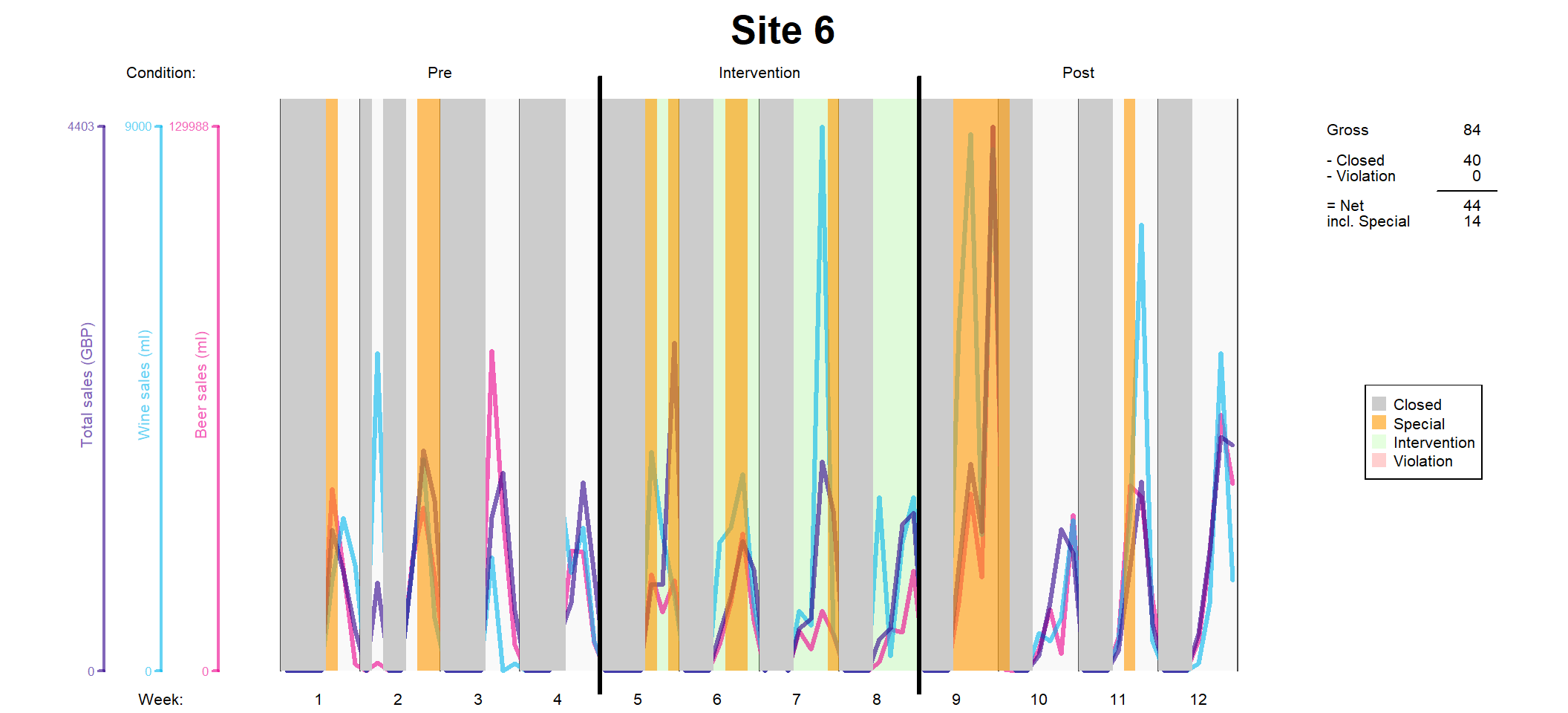

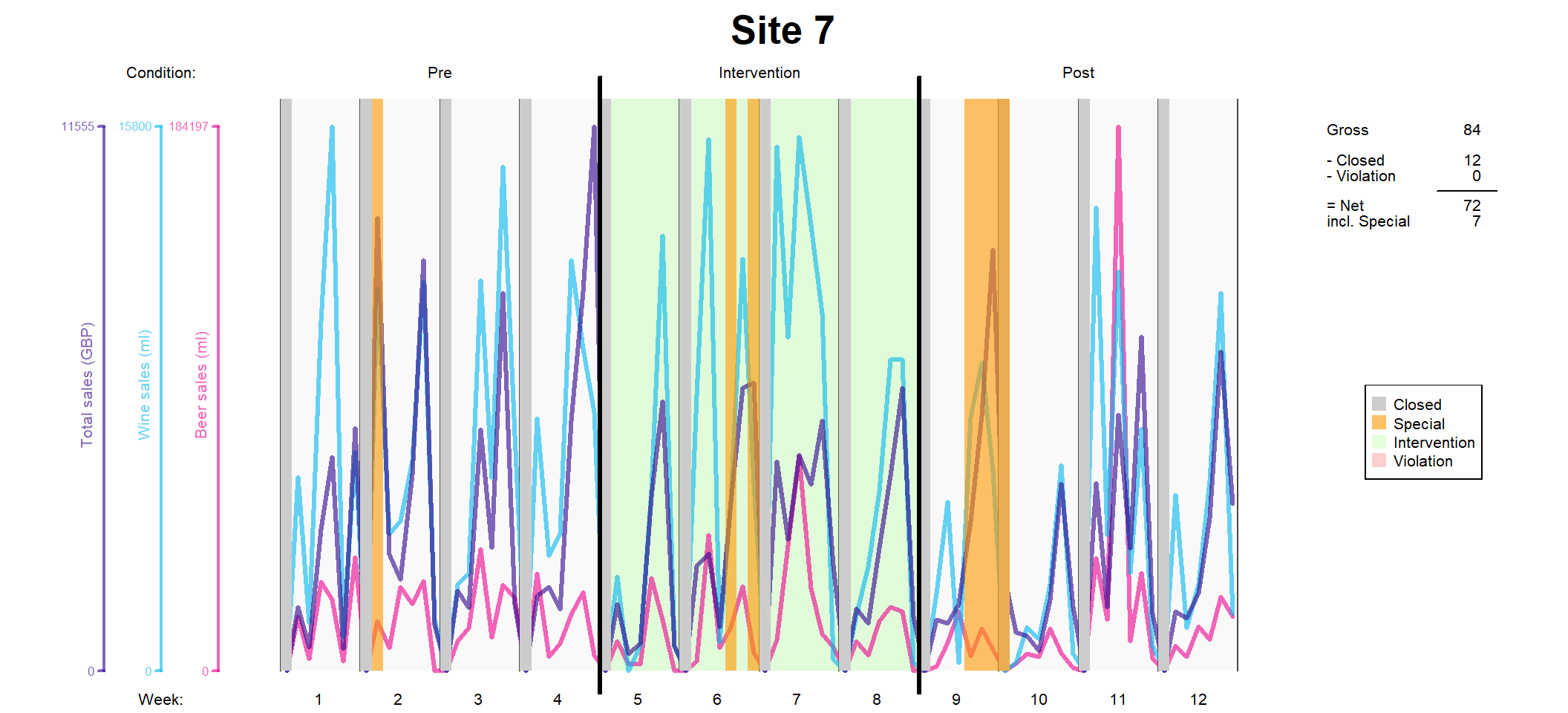

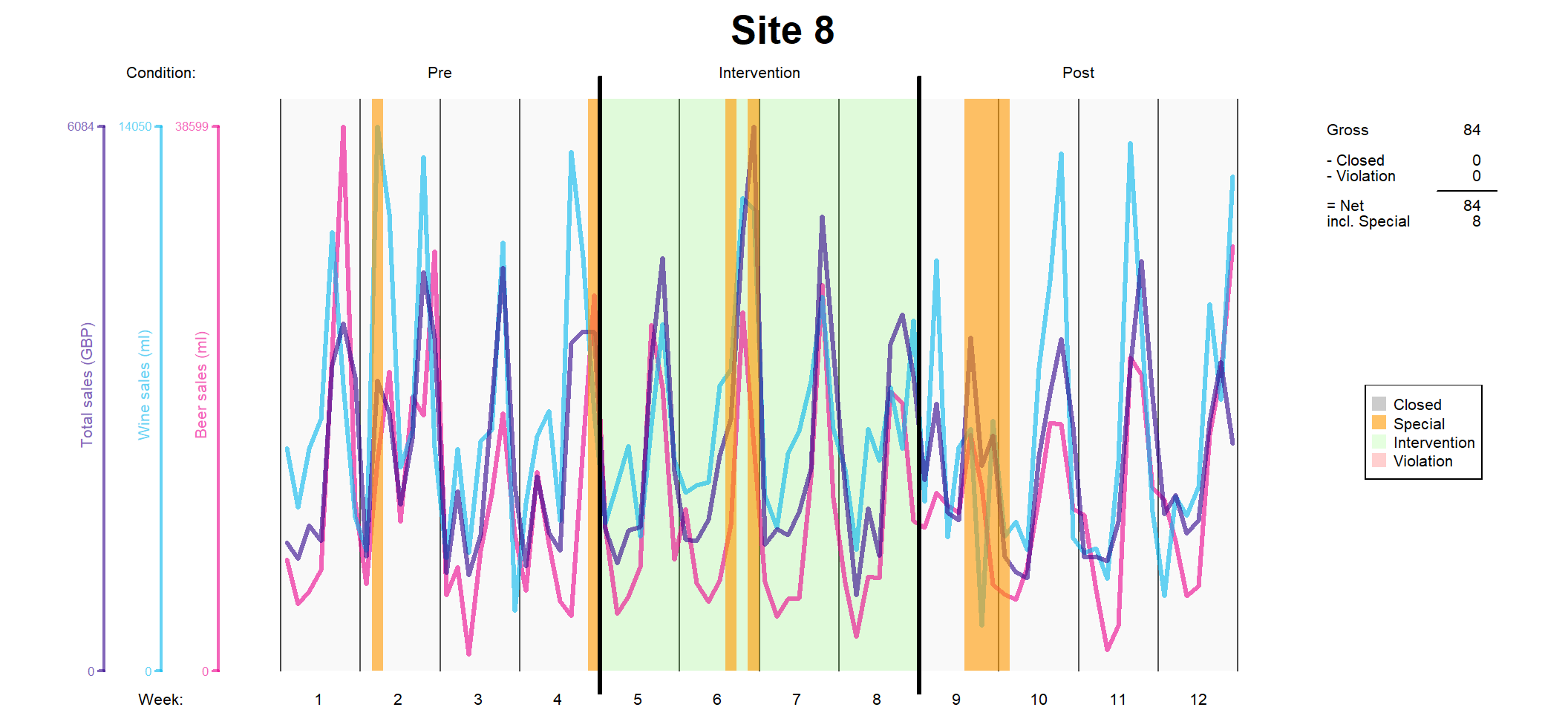

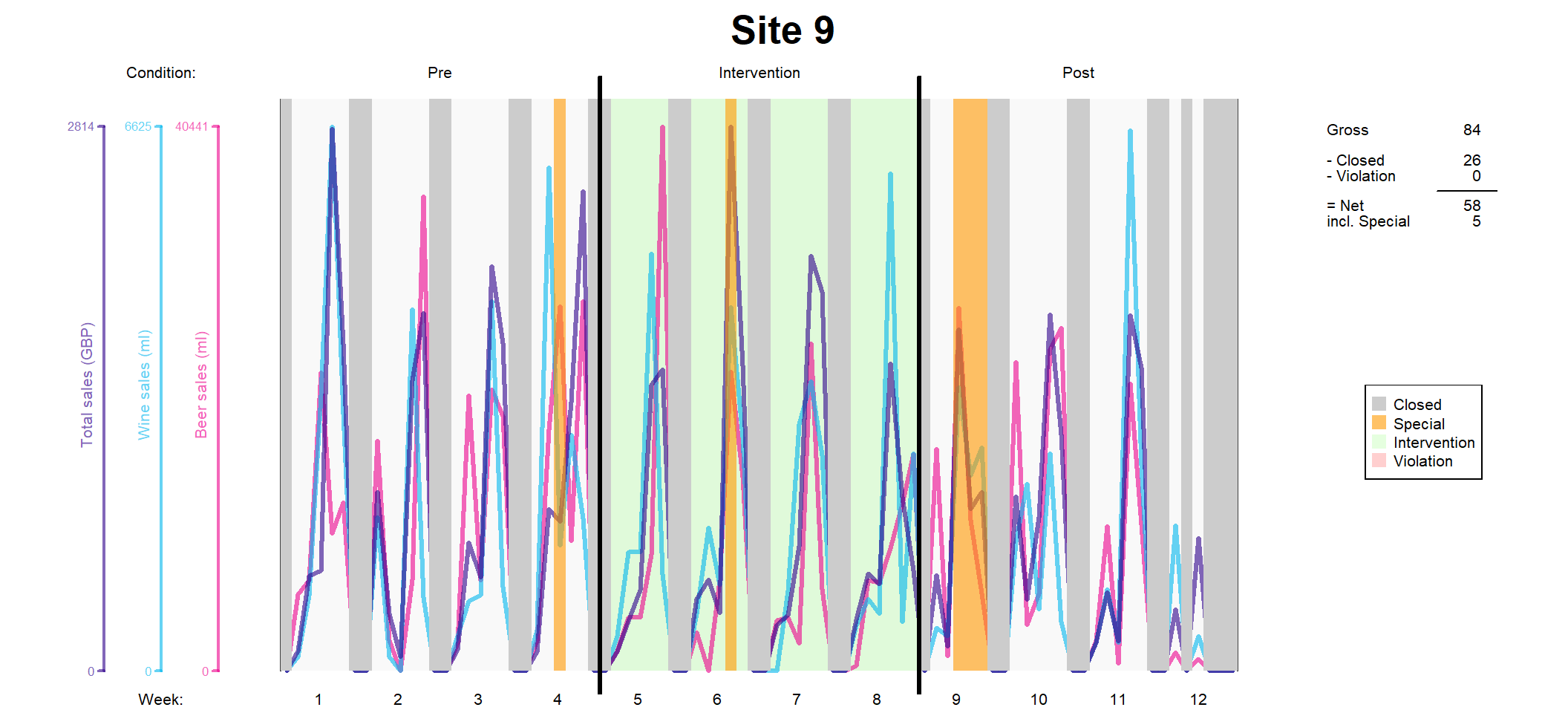

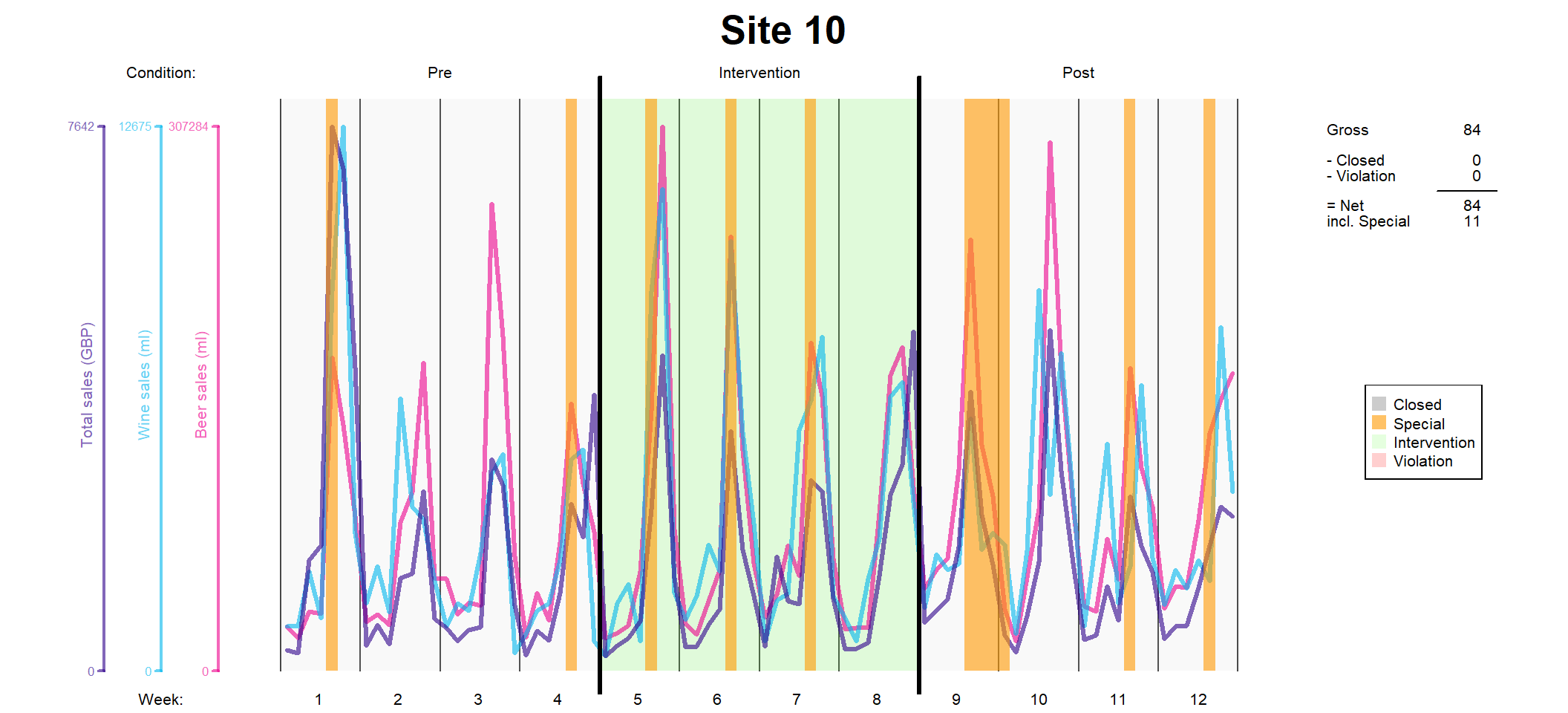

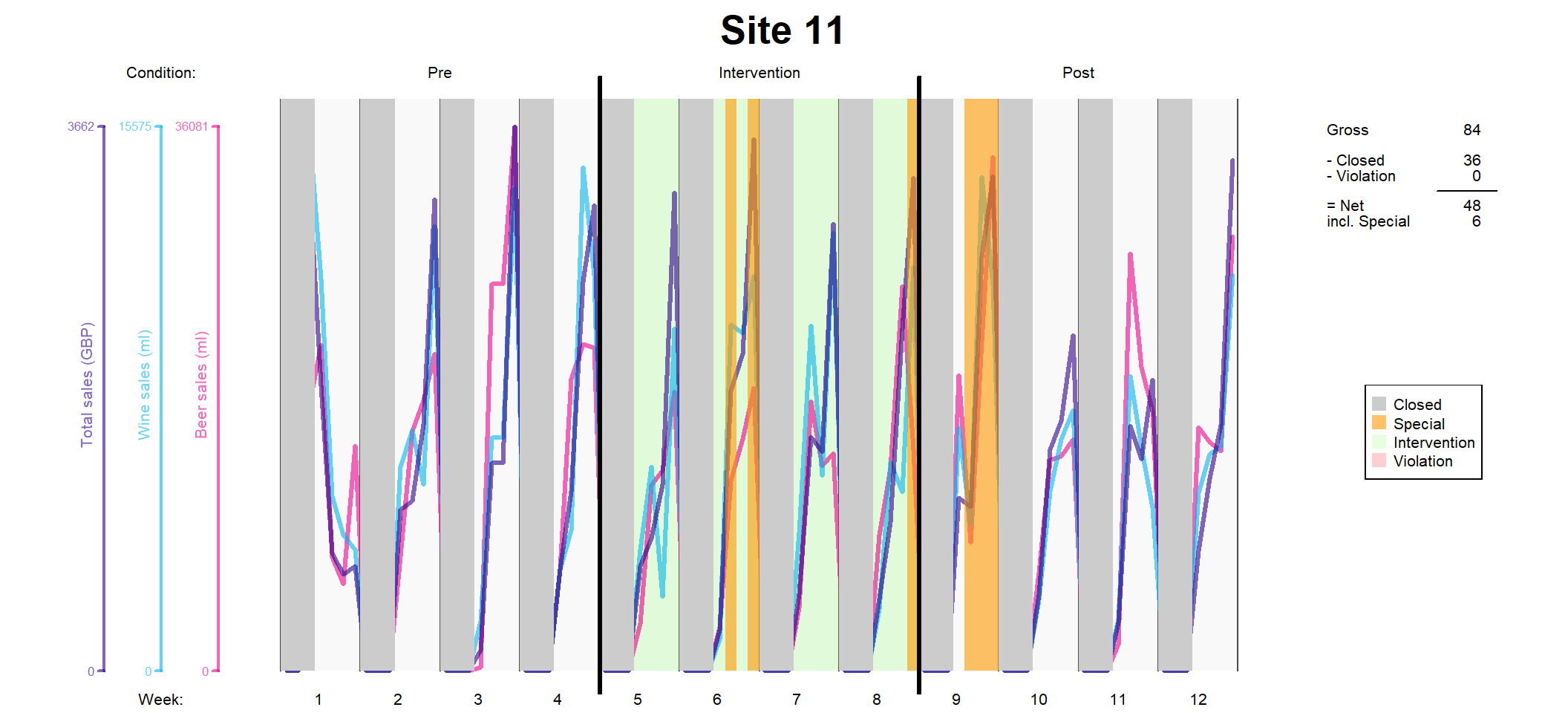

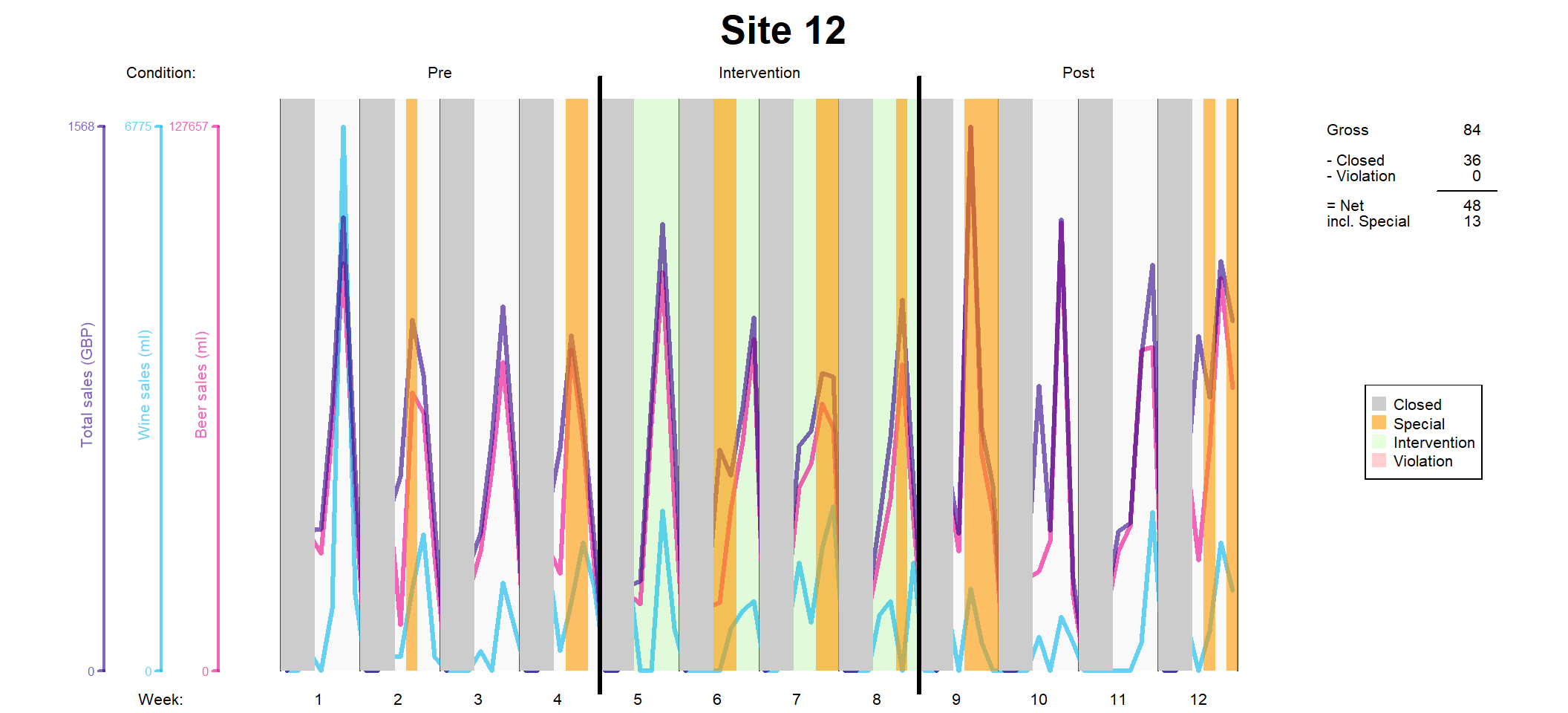

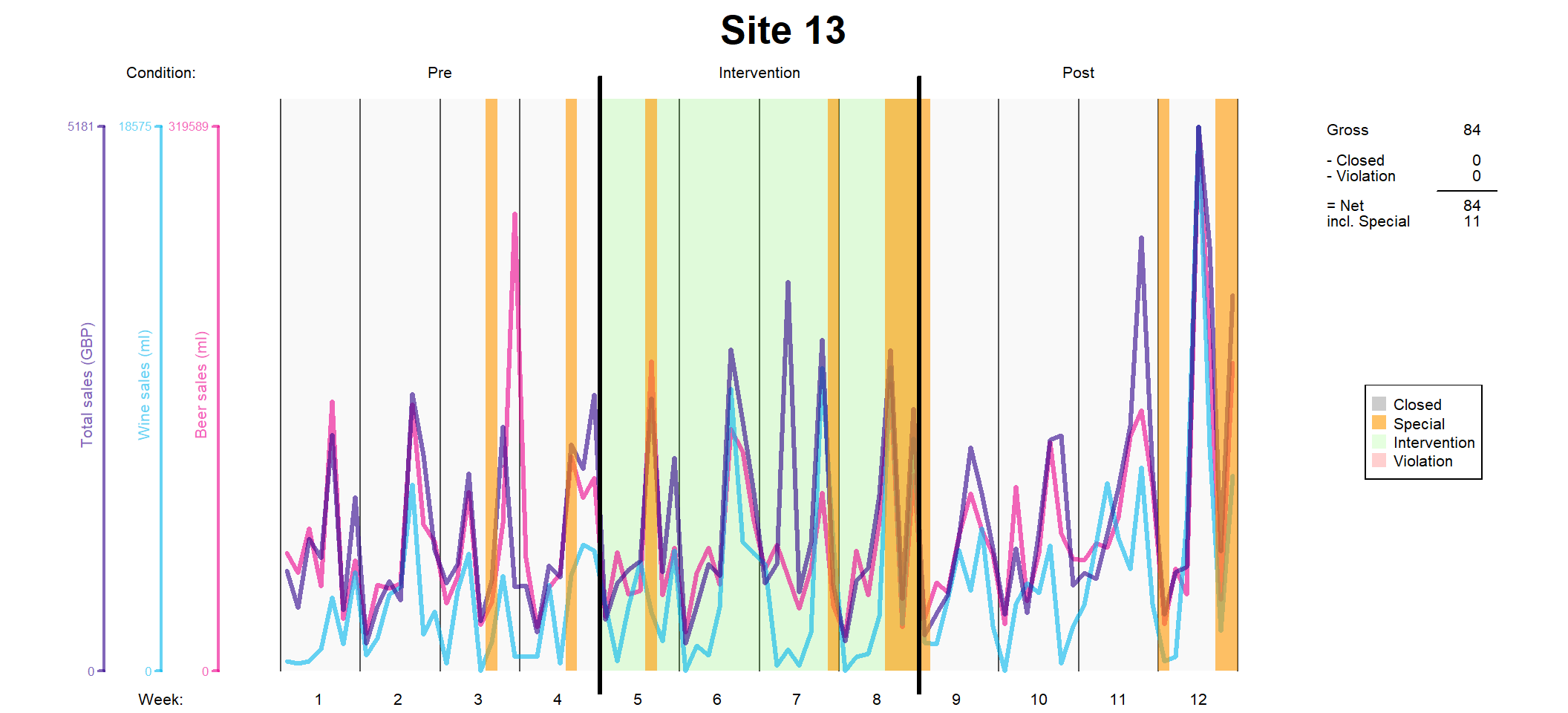
**

**Fig. B:** Change in daily volume of beer and wine sold (ml) with intervention

**
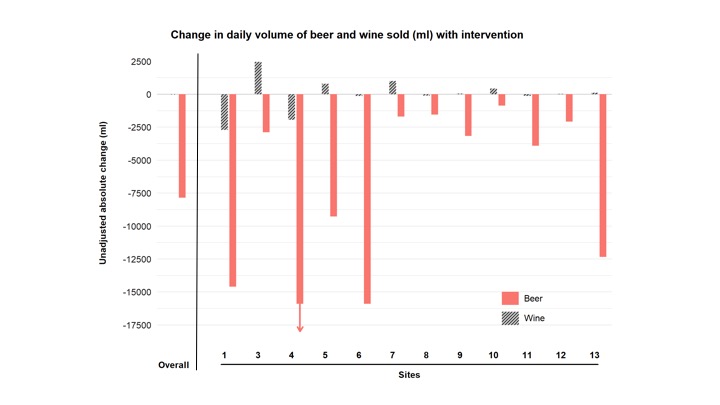
Note:** the arrow ( ) on the bar illustrating the volume of beer sold in Site 4 indicates that the value goes beyond the range of the axis
