# Supplementary material for: Impact on beer sales of removing the pint serving size: An A-B-A reversal trial in pubs, bars, and restaurants in England
Source: PLoS Med. 2024 Sep 17;21(9):e1004442. doi: 10.1371/journal.pmed.1004442 (PMC11407663; doi:10.1371/journal.pmed.1004442)
Supplement: S2 Appendix — (PDF) [file pmed.1004442.s002.pdf]

Cambridge, December 6, 2022

Dominique-L. Couturier, Ph.D.
Senior Research Associate

MRC Biostatistics Unit
University of Cambridge
East Forvie Building
Robinson Way
Cambridge CB2 0SR

'B' +44 7478 658 009
C8 dominique.couturier@mrc-bsu.cam.ac.uk

Dr. Eleni Mantzari
Behaviour and Health Research Unit
University of Cambridge
Cambridge CB2 0SR

Subject : **Sample size calculation [#20221011]**

Dear Eleni,

You are interested in defining the **number of sites** required to detect a decrease in beer sales induced by an intervention assuming

- the same ABA reversal design (set over three 4-weekly periods) as considered in your wine study for all sites,
- the same effect size as estimated in your wine study.

In this document, we briefly describe the results of the sample size calculation we performed.

/1/ Simulation parameters

On the 10/11/2022, you kindly shared the raw data of your wine study. These data also include daily beer sales, your primary outcome, for which a log normal model mixed model with

- **day of week**, (standardised) **time**, (standardised) **revenue** and (standardised) **temperature** as fixed effects,
- a random intercept and revenue slope per site,
- site heteroscedastic error terms,

i.e., a similar model as the one you chose for the wine study, seemed suitable.

To define the sample size on interest, we performed a simulation-based predictive power analysis [1,2] allowing to incorporate the uncertainty related to the target parameter as estimated in the wine study. We considered

- **H0** : $\beta_1 = 0$ versus **H1** : $\beta_1 < 0$,
  as statistical hypotheses where β_1 denotes the intervention fixed effect parameter,
- a 2.5% type error (often considered for one-sided tests),
- two estimators, the REML estimator of [3] and the GAMLSS estimator of [4].

We also assumed

— the same model as described above plus a dichotomous intervention predictor,
— the same parameters and parameter covariance as estimated on the beer data of your previous study,
— the same intervention parameter value and uncertainty as estimated on the wine data of your previous study.

/2/ Results

Figure 1 shows the type I error (y-axis) as a function of the number of sites (x-axis) and estimator (coloured lines), as estimated by our Monte Carlo simulation under **H0**. The horizontal black line corresponds to the α nominal value of 2.5% and the light grey rectangle corresponds to the Monte Carlo simulation error. We can note that the observed type I error of both estimators is slightly greater than the nominal value in most cases, suggesting that significance should be assessed with care when considering a model as complex as the one described in **Section 1**.

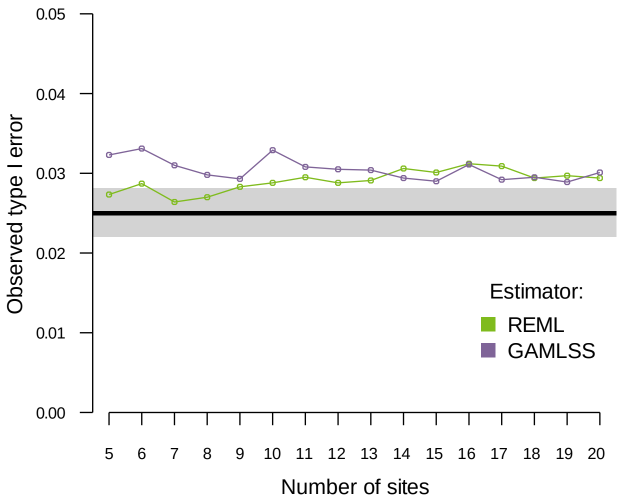

Figure 1 *: Estimated type I error (y-axis) as a function of the number of sites (x-axis) and estimator (coloured lines)*

Figure 2 shows the power (y-axis) as a function of the number of sites (x-axis) and estimator (coloured lines), as estimated by our Monte Carlo simulation under **H1**. We can note that

— the REML estimator seemingly leads to a greater power while often achieving a better type I error control,
— the power of the REML is above 0.85 for all considered number of sites.

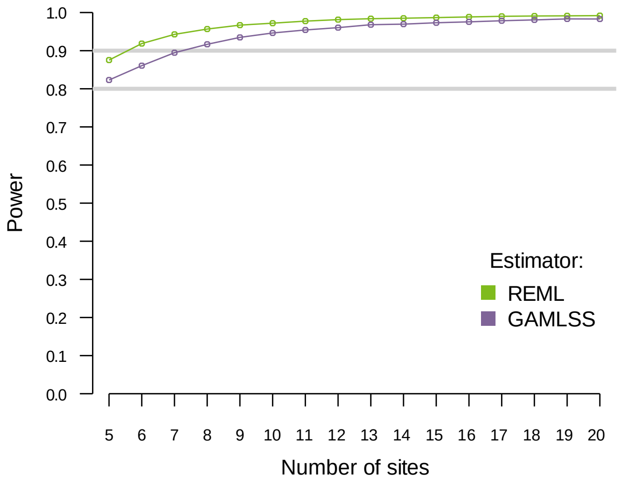

Figure 2 *: Estimated power (y-axis) as a function of the number of sites (x-axis) and estimator (coloured lines)*

/3/ Conclusion

The Monte Carlo simulation we considered to assess the operating characteristics of a trial considering the same design and (estimated) effect size as in the wine study, as well as the relationship between the different predictors of interest and your new primary outcome as noted in the your wine study showed that your beer study is already powered at the 85% level with a number of sites as low as 5.

We would suggest to consider a larger number of sites nevertheless due to the model complexity [5].

Let me know if you have questions.

Kind regards,

Dominique-Laurent Couturier

References

1/ Micheloud, C and Held, L. (2022). *Power Calculations for Replication Studies*, Stat. Science, https://doi.org/10.1214/21-STS828

2/ Spiegelhalter, D. J. and Freedman, L. S. (1986). *A predictive approach to selecting the size of a clinical trial, based on subjective clinical opinion.* Stat. Med., https://doi.org/10.1002/sim.4780050103

3/ Pinheiro, J.C. and Bates, D.M (2000), Mixed-Effects Models in S and S+, Springer, ISBN : 978-0-387-22747-4

4/ Robert A. Rigby, R.A., Stasinopoulos, M.D., Heller, G.Z. and De Bastiani, F. (2019), Distributions for Modeling Location, Scale, and Shape, Chapman Hall, ISBN : 9781032089423

5/ Bolker, B. (2022). *GLMM FAQ*. http://bbolker.github.io/mixedmodels-misc/glmmFAQ.html
