# Supplementary material for: Impact on beer sales of removing the pint serving size: An A-B-A reversal trial in pubs, bars, and restaurants in England
Source: PLoS Med. 2024 Sep 17;21(9):e1004442. doi: 10.1371/journal.pmed.1004442 (PMC11407663; doi:10.1371/journal.pmed.1004442)
Supplement: S3 Appendix — (DOCX) [file pmed.1004442.s003.docx]

**Appendix S3 – Additional Tables**

**Table A:** Heteroscedastic linear mixed model main results, estimating the volume (ml) of beer sold per day on the cubic root (n=12).

|  | Value | Std.Error | DF | t-value | p-value |
| --- | --- | --- | --- | --- | --- |
| (Intercept) | 30.617 | 2.350 | 779 | 13.027 | <0.00001 |
| INTERVENTION | -1.014 | 0.191 | 779 | -5.318 | <0.00001 |
| Monday | 0.279 | 0.372 | 779 | 0.751 | 0.45311 |
| Tuesday | -0.505 | 0.315 | 779 | -1.606 | 0.10871 |
| Wednesday | -0.185 | 0.341 | 779 | -0.544 | 0.58643 |
| Thursday | -0.697 | 0.286 | 779 | -2.437 | 0.01502 |
| Friday | 0.727 | 0.274 | 779 | 2.647 | 0.00827 |
| Saturday | 0.678 | 0.292 | 779 | 2.324 | 0.02041 |
| Sunday | -0.296 | 0.312 | 779 | -0.947 | 0.34383 |
| time.stand | 0.203 | 0.107 | 779 | 1.896 | 0.05830 |
| revenue.stand | 5.516 | 0.715 | 779 | 7.716 | <0.00001 |
| temp.stand | 0.087 | 0.111 | 779 | 0.777 | 0.43744 |
| special | -0.667 | 0.260 | 779 | -2.563 | 0.01056 |

**Table B –** Results of Sensitivity analysis 1: Modelling the primary analysis without controlling for other predictors

|  | **Value** | **Std.Error** | **DF** | **t-value** | **p-value** |
| --- | --- | --- | --- | --- | --- |
| (Intercept) | 22.298 | 1.825 | 840 | 12.216 | <0.00001 |
| INTERVENTION | -1.069 | 0.196 | 840 | -5.451 | <0.00001 |

**Table C -** Results of Sensitivity analysis 2: Intention-to-treat analysis including protocol violators

|  | **Value** | **Std.Error** | **DF** | **t-value** | **p-value** |
| --- | --- | --- | --- | --- | --- |
| (Intercept) | 31.213 | 2.241 | 830 | 13.930 | <0.00001 |
| INTERVENTION | -1.028 | 0.189 | 830 | -5.440 | <0.00001 |
| Monday | 0.319 | 0.373 | 830 | 0.853 | 0.39367 |
| Tuesday | -0.481 | 0.316 | 830 | -1.523 | 0.12806 |
| Wednesday | -0.055 | 0.336 | 830 | -0.164 | 0.87012 |
| Thursday | -0.713 | 0.283 | 830 | -2.515 | 0.01210 |
| Friday | 0.604 | 0.270 | 830 | 2.237 | 0.02558 |
| Saturday | 0.670 | 0.290 | 830 | 2.311 | 0.02110 |
| Sunday | -0.343 | 0.311 | 830 | -1.105 | 0.26946 |
| Study Day | 0.216 | 0.106 | 830 | 2.024 | 0.04325 |
| Daily revenue | 6.163 | 0.903 | 830 | 6.824 | <0.00001 |
| Temperature | 0.110 | 0.111 | 830 | 0.987 | 0.32373 |
| Special events | -0.695 | 0.259 | 830 | -2.688 | 0.00733 |

**Table D -** Results of Sensitivity analysis 3 - Modelling the primary analysis while considering the intervention predictor as a three-level factor

|  | **Value** | **Std.Error** | **DF** | **t-value** | **p-value** |
| --- | --- | --- | --- | --- | --- |
| (Intercept) | 30.905 | 2.262 | 829 | 13.664 | <0.00001 |
| INTERVENTION | -0.735 | 0.394 | 829 | -1.866 | 0.06246 |
| POST | 0.611 | 0.686 | 829 | 0.890 | 0.37366 |
| Monday | 0.287 | 0.376 | 829 | 0.764 | 0.44501 |
| Tuesday | -0.492 | 0.317 | 829 | -1.552 | 0.12113 |
| Wednesday | -0.092 | 0.337 | 829 | -0.273 | 0.78510 |
| Thursday | -0.709 | 0.283 | 829 | -2.507 | 0.01235 |
| Friday | 0.619 | 0.270 | 829 | 2.292 | 0.02218 |
| Saturday | 0.698 | 0.291 | 829 | 2.401 | 0.01656 |
| Sunday | -0.311 | 0.312 | 829 | -0.998 | 0.31851 |
| Study Day | -0.007 | 0.280 | 829 | -0.026 | 0.97937 |
| Daily revenue | 6.157 | 0.900 | 829 | 6.838 | <0.00001 |
| Temperature | 0.087 | 0.113 | 829 | 0.771 | 0.44076 |
| Special events | -0.687 | 0.259 | 829 | -2.656 | 0.00806 |

∗

**Table E -** Results of Sensitivity analysis 4 – Comparing aggregate period sales

|  | **A** | **B** | **B-A** | **100(B/A-1)** |
| --- | --- | --- | --- | --- |
| Site 1 | 41705 | 27105 | -14600 | -35% |
| Site 3 | 15568 | 12664 | -2903 | -19% |
| Site 4 | 74777 | 48786 | -25990 | -35% |
| Site 5 | 71141 | 61847 | -9293 | -13% |
| Site 6 | 29919 | 14006 | -15913 | -53% |
| Site 7 | 19149 | 17457 | -1692 | -9% |
| Site 8 | 12476 | 10918 | -1558 | -12% |
| Site 9 | 12522 | 9353 | -3169 | -25% |
| Site 10 | 88236 | 87364 | -872 | -1% |
| Site 11 | 17022 | 13100 | -3922 | -23% |
| Site 12 | 50649 | 48573 | -2076 | -4% |
| Site 13 | 84469 | 72120 | -12349 | -15% |
| AVERAGE | 43136 | 35274 | -7862 | -20% |

**Table F** Unadjusted mean number (sd) of beers sold per day according to serving size and study period and negative binomial analysis results (n=12)

| **Serving size** | **Non-intervention periods** | **Intervention period** | **Estimate** | **Std. Error** | **z value** | **Pr(>\|z\|)** |
| --- | --- | --- | --- | --- | --- | --- |
| 1/3 pints (189ml) | 0.44 (2.13) | 0.39 (1.76) | -0.07 | 0.2 | -0.36 | 0.72175 |
| 275ml bottle | 0.00 (0.06) | 0.01 (0.10) | 2.7 | 1.87 | 1.44 | 0.14896 |
| 1/2 pints (284ml) | 7.60 (10.8) | 8.01 (10.3) | 0.17 | 0.06 | 2.6 | 0.00937* |
| 330ml bottle | 10.1 (59.4) | 6.52 (23.8) | 0.02 | 0.09 | 0.21 | 0.83448 |
| 355ml bottle | 0.00 (0.00) | 0.01 (0.12) | 405.38 | 797437.11 | 0 | 0.99959 |
| 375ml bottle | 0.05 (0.32) | 0.03 (0.22) | -0.54 | 0.43 | -1.26 | 0.20653 |
| 2/3 pints (378ml) | 1.57 (5.92) | 78.5 (100.3) |  |  |  |  |
| 440ml bottle | 2.46 (10.4) | 2.79 (11.8) | 0.17 | 0.07 | 2.48 | 0.01312* |
| 500ml bottle | 2.79 (5.34) | 3.04 (6.47) | -0.01 | 0.09 | -0.11 | 0.91155 |
| 550ml bottle | 0.03 (0.27) | 0.03 (0.31) | -0.4 | 0.58 | -0.69 | 0.48743 |
| 568ml can | 0.01 (0.14) | 0.07 (0.39) | 2.39 | 0.65 | 3.66 | 0.00025* |
| Pints (568ml) | 63.1 (73.5) | 0.00 (0.00) |  |  |  |  |
| 660ml bottle | 0.00 (0.06) | 0.00 (0.00) | -20.02 | 13349.96 | 0 | 0.9988 |
| 750ml bottle | 0.03 (0.27) | 0.03 (0.17) | -0.03 | 0.51 | -0.07 | 0.94786 |

**Table G:** Heteroscedastic linear mixed model main results estimating the volume (ml) of wine sold per day on the cubic root (n=12).

|  | **Value** | **Std.Error** | **DF** | **t-value** | **p-value** |
| --- | --- | --- | --- | --- | --- |
| (Intercept) | 14.779 | 1.188 | 779 | 12.437 | <0.00001 |
| INTERVENTION | 0.344 | 0.163 | 779 | 2.109 | 0.03530 |
| Monday | -0.686 | 0.278 | 779 | -2.466 | 0.01387 |
| Tuesday | 0.183 | 0.238 | 779 | 0.77 | 0.44162 |
| Wednesday | 0.443 | 0.218 | 779 | 2.033 | 0.04241 |
| Thursday | 0.698 | 0.195 | 779 | 3.583 | 0.00036 |
| Friday | 0.288 | 0.2 | 779 | 1.444 | 0.14914 |
| Saturday | -0.134 | 0.255 | 779 | -0.525 | 0.59960 |
| Sunday | -0.793 | 0.274 | 779 | -2.89 | 0.00397 |
| Study Day | -0.36 | 0.09 | 779 | -3.993 | 0.00007 |
| Daily revenue | 3.42 | 0.39 | 779 | 8.762 | <0.00001 |
| Temperature | 0.128 | 0.095 | 779 | 1.355 | 0.17596 |
| Special events | 0.045 | 0.265 | 779 | 0.17 | 0.86469 |

**Table H:** Heteroscedastic linear mixed model main results estimating the daily revenue (£) on the log scale (n=12).

|  | **Value** | **Std.Error** | **DF** | **t-value** | **p-value** |
| --- | --- | --- | --- | --- | --- |
| (Intercept) | 7.170 | 0.215 | 780 | 33.399 | <0.00001 |
| INTERVENTION | -0.052 | 0.025 | 780 | -2.080 | 0.03784 |
| Monday | -0.510 | 0.042 | 780 | -12.075 | <0.00001 |
| Tuesday | -0.452 | 0.046 | 780 | -9.882 | <0.00001 |
| Wednesday | -0.408 | 0.030 | 780 | -13.384 | <0.00001 |
| Thursday | -0.153 | 0.030 | 780 | -5.136 | <0.00001 |
| Friday | 0.421 | 0.026 | 780 | 15.962 | <0.00001 |
| Saturday | 0.713 | 0.027 | 780 | 26.550 | <0.00001 |
| Sunday | 0.389 | 0.039 | 780 | 10.083 | <0.00001 |
| Study Day | 0.010 | 0.014 | 780 | 0.682 | 0.49548 |
| Special events | 0.071 | 0.040 | 780 | 1.787 | 0.07434 |
| Temperature | 0.034 | 0.015 | 780 | 2.351 | 0.01895 |
