# Supplementary material for: Impact on beer sales of removing the pint serving size: An A-B-A reversal trial in pubs, bars, and restaurants in England
Source: PLoS Med. 2024 Sep 17;21(9):e1004442. doi: 10.1371/journal.pmed.1004442 (PMC11407663; doi:10.1371/journal.pmed.1004442)
Supplement: Attachment — Submitted filename: Pints study_response to reviewers FINAL.docx [file pmed.1004442.s005.docx]

Dear Editor,

In response to your email dated 10^th^ April 2024 regarding our manuscript entitled “Impact on beer sales of removing the pint serving size: an A-B-A reversal trial in pubs, bars and restaurants in England”, please find below our responses to the Editor (9 points) Reviewer 1 (12 points) Reviewer 2 (6 points) and Reviewer 3 (9).

We are grateful for the opportunity to address these many helpful points to generate a stronger manuscript, with changes to the manuscript highlighted in yellow.

Note: we have re-run our analysis using updated versions of R and R packages. Consequently, some estimates have changed. These differences are negligible, and do not change the conclusions in any way.

Yours Faithfully,

The authors

**Requests from the editors:

1. Please include line numbers in your revised manuscript, ideally not starting from 1 with each new page.**

This has now been done.

**2. Please cite the reference numbers in square brackets. Citations should precede punctuation.**

This has now been amended. **3. Abstract: Please report your abstract according to CONSORT for abstracts, following the PLOS Medicine abstract structure (Background, Methods and Findings, Conclusions) (see per previous study “Impact on wine sales of removing the largest serving size by the glass: An A-B-A reversal trial in 21 pubs, bars, and restaurants in England’ -**
The abstract has now been amended according to the above.

**4. At this stage, we ask that you include a short, non-technical Author Summary of your research to make findings accessible to a wide audience that includes both scientists and non-scientists. The Author Summary should immediately follow the Abstract in your revised manuscript. This text is subject to editorial change and should be distinct from the scientific abstract. Ideally each sub-heading should contain 2-3 single sentence, concise bullet points containing the most salient points from your study. In the final bullet point of ‘What Do These Findings Mean?’, please include the main limitations of the study in non-technical language. Please see our author guidelines for more information:** [**https://eur03.safelinks.protection.outlook.com/?url=https%3A%2F%2Fjournals.plos.org%2Fplosmedicine%2Fs%2Frevising-your-manuscript%23loc-author-summary&data=05%7C02%7Ctm388%40medschl.cam.ac.uk%7Cc6a6b3cd99244628a75908dc59589d6c%7C49a50445bdfa4b79ade3547b4f3986e9%7C1%7C0%7C638483484346176241%7CUnknown%7CTWFpbGZsb3d8eyJWIjoiMC4wLjAwMDAiLCJQIjoiV2luMzIiLCJBTiI6Ik1haWwiLCJXVCI6Mn0%3D%7C0%7C%7C%7C&sdata=6oJzv6luc0BZrxdx5TQdlYicoiqkFxYftwecwfTIAqQ%3D&reserved=0**](https://journals.plos.org/plosmedicine/s/revising-your-manuscript#loc-author-summary)**.**

The author summary has now been added (page 3):

***Author Summary***

***Why was the study done***

- *Removing the largest serving size of wine by the glass (usually 250ml) reduces the volume of wine sold in licensed premises.*
- *It is unknown whether removing the largest serving size of other alcohol drinks, such as beer, has a similar effect.*

***What did the researchers do and find***

- *We asked 13 licensed premises in England to remove the offer of their largest serving size of draught beer (one imperial pint – 568ml) from available options for four weeks. We compared the total volume of beer sold during the intervention period to that sold during the non-intervention periods.*
- *Removing the largest serving size for draught beer (the imperial pint) reduced the daily mean volume of beer sold by 9.7%.*

***What do these findings mean***

- *This intervention merits consideration for inclusion in alcohol control policies.*
- *The findings are limited due to a lack of assessment of all alcoholic drinks sold in participating licensed premises, which would have allowed for consideration of whether people may have fully compensated for their reduced beer consumption by drinking other alcoholic drinks.*

**5. Please complete the CONSORT checklist and ensure that all components of CONSORT are present in the manuscript. When completing the checklist, please use section and paragraph numbers, rather than page numbers.**

The CONSORT checklist was uploaded with the original submission. We will ensure it also accompanies the resubmission.
 **6. Discussion: please present and organize the Discussion as follows: a short, clear summary of the article's findings; what the study adds to existing research and where and why the results may differ from previous research; strengths and limitations of the study; implications and next steps for research, clinical practice and/or public policy implications; followed by a one-paragraph conclusion. Please remove all subheadings within your Discussion.**

The Discussion has now been amended to adhere to the above.

**7. Financial Disclosure: The funding statement should include: specific grant numbers, initials of authors who received each award, URLs to sponsors’ websites. Also, please state whether any sponsors or funders (other than the named authors) played any role in study design, data collection and analysis, the decision to publish, or preparation of the manuscript. If they had no role in the research, include this sentence: “The funders had no role in study design, data collection and analysis, decision to publish, or preparation of the manuscript.”**

The Funding section has been amended to conform to the above. **8. Supplementary materials: Please note that supplementary materials are not checked and will be posted as supplied by the authors. Therefore, please double check. Please cite your Supporting Information as outlined here:** [**https://eur03.safelinks.protection.outlook.com/?url=https%3A%2F%2Fjournals.plos.org%2Fplosmedicine%2Fs%2Fsupporting-information&data=05%7C02%7Ctm388%40medschl.cam.ac.uk%7Cc6a6b3cd99244628a75908dc59589d6c%7C49a50445bdfa4b79ade3547b4f3986e9%7C1%7C0%7C638483484346182609%7CUnknown%7CTWFpbGZsb3d8eyJWIjoiMC4wLjAwMDAiLCJQIjoiV2luMzIiLCJBTiI6Ik1haWwiLCJXVCI6Mn0%3D%7C0%7C%7C%7C&sdata=L6xocgPUyUjWnVPNBQA9z08bDI%2F4i4WhVR%2BESDMV%2F%2B8%3D&reserved=0**](https://journals.plos.org/plosmedicine/s/supporting-information) **- Please note you may use almost any description as the item name of your supporting information as long as it contains an "S" and number. For example, “S1 Appendix” and “S2 Appendix,” “S1 Table” and “S2 Table. Please ensure each supplementary material has a call out (link) from your main manuscript.**

The supporting information has been renamed to S1 Appendix **9. To help us extend the reach of your research, please provide any Twitter handle(s) that would be appropriate to tag, including your own, your coauthors’, your institution, funder, or lab.**

Please use the following twitter handle: @behavchangedsgn

**Comments from the reviewers:

Reviewer #1:**

**Thanks for the opportunity to read your manuscript. My role is statistical reviewer, so I have focused on the design, data, and analysis that are presented. I have put general comments first, followed by questions relevant to a specific section of the manuscript (with a page/paragraph reference).

This study examines the effectiveness of removing 'pint' (568 mL) serving sizes from licenced premises, replacing with a smaller size serving (2/3rds of a pint). Thirteen sites were recruited for the study. The volume of beer and cider (draught and bottled) was the primary outcome, with secondary outcomes including size-specific volume of beer/cider, daily volume of wine, and daily revenue. Data was collected over 12 week, with a 4 week baseline period, 4 weeks of intervention (pints not available), and 4 weeks of withdrawal of intervention. Data was collected through the same time-period across all sites, and fidelity to the intervention was checked at all sites.
The main analysis used an extension of liner mixed models to allow for site-specific heterogeneity in variance. The main outcome was transformed (^1/3), with parameters in the model for study period, day of week, day of study, daily revenue, temperature, and special events. Adding daily revenue as a covariate means the main outcome for the study from this analysis can be thought of as beer/cider volume proportional to overall sales. Random intercepts were included for sites, and a random slope term for overall revenue of each site.

Several sensitivity analyses were considered, looking at the effect of removing covariates from the main analysis, data from sites that were not adherent, allowing baseline and reversal periods to have different levels, and an analysis that directly compared total volume of beer/cider aggregated to either no-intervention or intervention. There was a reduction in the volume of beer and cider sold during the intervention period, and with a 5% decline in daily revenues and a small increase in wine sold. The treatment effect from these sensitivity analyses was close to that seen in the main analysis.

1. There was a low response rate - does this affect the generalisability of the findings?**

We thank the reviewer for this observation. We have now added this possibility to the manuscript:

*Second, caution is needed in generalising these findings. The low response rate to an invitation to participate in the study might have resulted in sampling bias, meaning that participating premises may not have been representative of typical licensed premises in England. (page 15, lines 658-662)*
 **2. P9. Is 'wine' just table wine or does it include fortified wines?**

We did not include sales of fortified wines in our analysis. We have clarified this in the manuscript:

*A heteroscedastic linear mixed model analysis, similar to that used for the primary analysis (i.e. using the fixed and random effects) to estimate the daily volume of table wine (excluding fortified wines) sold according to study period (A vs B). (page 10, lines 432-433)* **3. P12, Paragraph 1. In the methods it is mentioned that all sites passed the fidelity checks - this seems to contradict this paragraph.**

All premises passed the fidelity checks, meaning that on the days these checks were conducted they were not found to be selling pints during the intervention period. However, we conducted checks only on one occasion during the intervention period and again once during return to the non-intervention period to ensure all premises had brough back the offer of pints. These checks are useful in ensuring that any observed protocol violations are addressed immediately. However, as there was still the possibility of protocol violations on days when checks were not conducted, sales data were also checked to ensure no pints were sold during the intervention period. From this inspection, we found that one premises had violated the protocol.

We have now clarified this in the manuscript:

*…No premise failed any fidelity checks but given these were conducted only once following each reversal, sales data were also checked for any protocol violations. (page 9, 373-374)*

**4. P13, Table 3. I would consider some adjustments to this table. Most of the information presented here about covariates could be safely moved to an appendix. I would also consider presenting just the estimate, p-value, and 95% CI here. The intercept is also superfluous to the main research questions. Is it also possible to present a back-transformed treatment effect estimate (with CL) in both the table and the main results? This would be ideal for all results where the outcome was transformed before the analysis.**

Thank you for your suggestions. We agree that much of the data in this table was not of direct interest and have now moved it to supplementary material (Figures S1a to S1m). As suggested, we now present the table (page 12) with covariate coefficients, standard errors, DF and t values removed, and with values on the original scale. We chose to retain the intercept as it allows readers to define the (average) beer sales outside the intervention period and to deduce the relative effect of the intervention by hand:

***Table 3:*** *Heteroscedastic linear mixed model main results, estimating the volume (ml) of beer sold per day on the transformed (cube root) scale, on the original scale and on the relative scale, covariate coefficients shown in Supplementary Table S1 (n=12). Rounding is to two decimal places.*

|  | ***Intercept*** | ***Intervention*** | | | |
| --- | --- | --- | --- | --- | --- |
| ***Scale*** | ***Estimate*** | ***Estimate*** | ***Lower 95% CI*** | ***Upper 95% CI*** | ***p-value*** |
| *Transformed (cube root)* | *30.62* | *-1.01* | *-1.39* | *-0.64* |  |
| *Original (ml)* | *29,126.66* | *-2,769.21* | *-4,188.46* | *-1,577.75* | *<0.00001* |
| *Relative (%)* | *100.00* | *-9.66* | *-13.52* | *-6.08* |  |

**5. Supplementary appendices. The titles/captions for the tables should have information about the type of sensitivity analysis in each case rather than just 1, 2, 3 etc.**

We have now changed the titles for the tales in the supporting information:

***Table S2 –*** *Results of Sensitivity analysis 1: Modelling the primary analysis without controlling for other predictors*

***Table S3 -*** *Results of Sensitivity analysis 2: Intention-to-treat analysis including protocol violators*

***Table S4 -*** *Results of Sensitivity analysis 3 - Modelling the primary analysis while considering the intervention predictor as a three-level factor*

***Table S5 -*** *Results of Sensitivity analysis 4 – Comparing aggregate period sales*

**6. The study has a pre-registered protocol and statistical analysis plan, these match the current manuscript.**

We thank the reviewer for taking the time to check. **7. P6, Paragraph 2. This is a comment and requires no response - I am absolutely delighted to see a mention of the strange names for beer glass sizes from South Australia. They are a complete aberration. As a frequent visitor to Adelaide I have been caught out many times by ordering a 'Schooner' (425mL in my hometown, 3/4 of a pint) and ending up with a disappointing sized glass of beer (285mL, 1/2 pint).**

We thank the reviewer for this comment and observation.

**8. P10, Paragraph 5. Just to check - does 'heteroscedastic LMM" mean that a different constraint was allowed for each site in residual variance? i.e. is this similar to what is done with the 'group' option in proc mixed in SAS or with the galamm package in R?**

In this instance, a different residual variance was permitted for each site and day of week. We fit the model using the lme() function in the nlme package in R, a package that is imported by galamm. In the galamm package article on heteroscedastic LMM, (https://lcbc-uio.github.io/galamm/articles/lmm_heteroscedastic.html), the authors give an example where the results from their package’s model fit and the lme() model fit are indeed identical. We now state our use of both package and software in the manuscript, thank you.

**9. P10, Paragraph 6. What checks of model fit were made?
What software was used for the analysis?**

Missed (linear or non-linear) trends and heteroscedasticity were tracked by looking at the distribution of the standardised residuals as a function of the fitted values and of different predictors, like maximum daily local temperature, day of the week, time in days from start of the study, daily total revenue, interventions and sites. These analyses suggested analysing the data on the cube root scale and amending the model to allow the residual variance to be a function of sites and days of the week.

The effect of outliers was investigated by assessing their effect on our conclusions. We therefore compared results of the restricted maximum likelihood with results obtained when

•	discarding observations corresponding to a (robust) weight smaller than 0.9 when fitting the standardised residuals as a function of the fitted values by means of a robust MM-estimator (function robustbase::lmrob() in R)

•	down-weighting the observations detected as outliers by means of a robust linear mixed model (function robustlmm::rlmer() in R)

These analyses led to similar intervention effect conclusions.

The effect of the assumed random effect structure was investigated by comparing conclusions obtained with fits considering alternative random effect structure. All analyses led to similar conclusions related to the intervention effect. The random effect structure leading to the best model check was preferred.

We used the R Statistical Software, version 4.4.0. This information has been added to the manuscript:

*…via the function lme of the nlme package of the R Statistical Software (version 4.4.0) (page 9, lines 395-396)*

**10. P10, Paragraph 7. I follow the rationale for most of these sensitivity analyses, except for #4. What was assumption being tested with aggregating the data and using a paired t-test?**

The aim of sensitivity analysis #4 is to examine the results of an alternative model, far simpler than the one considered above (no covariate adjustment and aggregated data per site, for example) and therefore relying on fewer model assumptions (paired t-test versus heteroscedastic linear mixed model), with the rationale that obtaining similar conclusions using a different modelling approach would strengthen our confidence in the results of the primary analysis. This is not obvious from reading the manuscript, so we have now made this clearer:

*Like sensitivity analysis 1, this model does not control for other predictors. Furthermore, the model relies on fewer assumptions than the primary analysis, suggesting that the conclusions of the primary analysis are not dependent on its assumptions*. (page 10, lines 425-426)

**11. P11, Paragraph 5. Did the negative binomial model just include a parameter for day of study, or did it include similar parameters to the main analysis? Was a negbin model used for every possible serving size?**

Yes, the negative binomial model contained parameters for intervention, revenue (standardised) and time (standardised), and was undertaken for every serving size.

**12. Figure 2. Instead of the 'dynamite' plot presented here, I'd consider plotting as much of the data as possible, e.g. is it possible to construct a panel graph showing the time series of primary outcome across all the study in each site? I think with the type of model used, it should also be possible to estimate a 'counterfactual' estimate of beer/cider volume sold if the intervention was not implemented (similar to an interrupted time series).**

Thank you for the suggestion of showing more granular data. We now present time series plots of sales across the study for each site individually in Figures S1a to S1m of our supplementary material and alert the reader to these plots in the main manuscript under “Primary outcome analysis”.

Instead of overlaying these different time series in a single figure, which resulted in a confusing display due to the number of sites, their varying sale levels and sales variability, we chose to:

- show the data for each site separately. Such plots also allowed us to provide additional information like the presence of special events, time at which venues were closed as well as wine and total sales.
- keep Figure 2 in the main manuscript as it provides a standardised comparison of the intervention effect per site (and related uncertainty) through the use of relative changes (and confidence intervals) on top of the overall average intervention effect.

**Reviewer #2:**

**The authors have presented an intervention study with a clear rationale robust methodology. I applaud the authors use of transparent research methods, having registered their work with the open science framework prior to publication. The authors are following up on their previous work, examining whether reducing the servicing size of wine reduced consumption but now looking at beer products. They find a similar result, lowering serving sizes does result in decreased consumption. The article is of high quality and answers an important question - while the similarities to their previous work are undeniable this is a strength of the article rather than a limitation - they are building on their prior work in a meaningful way.**

We would like to thank the reviewer for their positive feedback.

**There are some points I would like to see the authors address ahead of publication:**

**1.      First sentence appears to have a citation error (1), (2). Should be (1,2).**

Thank you for the observation. This has now been corrected.

**2.      "Licensed premises in Western Australia have undergone a process by which a pint-drinking culture has been replaced by a schooner-drinking one, to tackle increasing costs and adhere to government efforts to reduce alcohol consumption (32-34)." - the references used here are two media publications and a Wikipedia article, the authors should use higher quality empirical evidence when discussing drinking trends and their causes. This point does not add anything of value to the manuscript and could be cut.**

We thank the reviewer for this observation. We agree that Wikipedia is not necessarily the most reliable source. We have therefore removed the relevant reference and have included an additional news article instead, which briefly mentioned that many venues in Western Australia are making schooners their biggest size. We have also revised the manuscript to acknowledge that the information derives from news articles due to the lack of relevant scientific research:

*As suggested by news reports, licensed premises in Western Australia have undergone a process by which a pint-drinking culture has been replaced by a schooner-drinking one, to tackle increasing costs and adhere to government efforts to reduce alcohol consumption. We are unaware of any relevant scientific research that corroborates this. (page 4, lines 206-209)*

**3.      "A simulation-based predictive power analysis" - could the authors go into more detail here, what was the exact method used, the statistical software, packages/commands. The reader should be able to replicate this process from the information provided in the text.**

We agree that the information provided in the manuscript do not allow the reader to get a precise idea of the process used to define our number of sites. We chose to

- keep the rather ‘non-technical’ description of the sample size calculation currently available in the manuscript as such as it provides a basic understanding of how the number of sites was defined
- add to the supplemental material the ‘Sample size calculation report’ related to this study as it provides the relevant technical detail allowing the reader to replicate the process (references to the concept and practicalities of the predictive power approach [#1 and #2] and to the R packages used, namely nlme [#3] and gamlss [#4]) are provided)

A reference to this new supplemental material has been added to the manuscript:

*The sample size calculation report providing a more thorough description of the process used to define the number of premises is available as supplemental material (S2 Appendix). (page 7, 293-294)*

**4.      "Thirteen licensed premises were recruited from 1740 contacted in six geographical areas of England, a recruitment rate of 0.75%." - this is incredibly low, what were the reasons that the other 1727 venues were not recruited - was this part of the recruitment strategy (e.g. contacting all venues and then recruiting the first ones that came back) or was this due to 1727 venues saying no?**

This is indeed a low response rate; lower than the response rate we observed in our previous similar study focusing on wine. The reason the majority of the contacted premises didn’t participate was that they did not respond to our invitation email: of 1740 contacted, 1712 didn’t respond. Figure 1 has been revised to show this information. We expect this to have been related to the nature of the intervention and the request to remove the sale of pints for the study. Only 28 responded expressing an interest. As can be seen in Figure 1, of those 28, 6 declined participation, one was ineligible and 8 did were unresponsive to further attempts at communication.

We acknowledge that the low response rate might limit the generalisability of the findings so we have now added the following to the manuscript:

*Second, caution is needed in generalising these findings. The low response rate to an invitation to participate in the study might have resulted in sampling bias, meaning that participating premises may not have been representative of typical licensed premises in England. (page 15, lines 658-662)*

**5.      "ABV for beer of 4.6%" - this brings up an important point, could people have ordered a stronger beer to compensate for the smaller size? In Australia, low mid and full-strength beers are available for purchase, are these available in the UK? Could this intervention move someone from a mid to full strength beer?**

This is an important comment. Although we cannot empirically refute this concern as we did not extract data relating to the individual beers sold during each study period, we believe that the possibility that customers compensated for the reduced serving sizes by drinking beers higher in %ABV is minimal for the following four reasons:

1. The majority of premises (10/13) offered beers with vary small variations in the % ABV, which ranged between 4% and 5%. Two premises also offered a few beers with 6% ABV, although most their range was again between 4% and 5%. Only one premises offered some options with higher ABV (up to 12%).
2. Beers lists did not change during the study
3. None of the premise managers or owners interviewed at the end of the study mentioned any changes in the drinking patterns of their customers during the intervention period relating to beer strength.

We have added the following information to the Discussion:

*It is also not known whether customers compensated for the removal of pints by drinking stronger beers i.e. those higher in percentage alcohol by volume (% ABV). We judged this unlikely given there were very small variations in the % ABV of beers sold by premises, with most options ranging from 4% to 5%. Also, beers lists did not change during the study and none of the premises managers interviewed at the end of the study reported any changes in the drinking patterns of their customers during the intervention period relating to beer strength (page 15, lines 652-658)*

**6.      "The pint is by far the most popular serving size in the UK (or England?)" - the authors should choose whether it is the UK or England they are referring to.**

We thank the reviewer for observing this mistake. It should read in the UK, so the information about England has been deleted:

*The pint is by far the most popular serving size in the UK ~~(or England)~~ (page 16, line 673)*
 **Reviewer #3:**

**This is a great, well-written paper on an important topic. I enjoyed reading it. I do have some comments which I think will improve this manuscript.**

We thank the reviewer for this positive feedback.

**Abstract**

**1. I don't think this is the first study to assess the impact of reducing serving size of beer on sales in a licensed premises. I think that is also examined in this published paper, though admittedly it is observed sales:** [**https://eur03.safelinks.protection.outlook.com/?url=https%3A%2F%2Fonlinelibrary.wiley.com%2Fdoi%2F10.1111%2Fadd.14228&data=05%7C02%7Ctm388%40medschl.cam.ac.uk%7Cc6a6b3cd99244628a75908dc59589d6c%7C49a50445bdfa4b79ade3547b4f3986e9%7C1%7C0%7C638483484346188727%7CUnknown%7CTWFpbGZsb3d8eyJWIjoiMC4wLjAwMDAiLCJQIjoiV2luMzIiLCJBTiI6Ik1haWwiLCJXVCI6Mn0%3D%7C0%7C%7C%7C&sdata=P5tYqfldhcQ2MAhq%2Fu8wOIk18tV9icnsRJHvNWErHLw%3D&reserved=0**](https://onlinelibrary.wiley.com/doi/10.1111/add.14228)**. Yours is the first study that I am aware of to do so using objective sales data. This study is still very important and extends the prior findings considerably, but this sentence should be toned down.**

We thank the reviewer for the opportunity to clarify. We are aware of the study the reviewer mentions and have indeed mentioned it in the Introduction. Although it was conducted in a pub setting, the context was semi-naturalistic. The pub was controlled by researchers and the range of available servings sizes was artificial. It was determined by researchers, rather than changes being made licensed premises’ existing ranges. Also, assessments were made during a single drinking occasion. Our study is the first we are aware of which has been conducted under real-world conditions, with changes made to premises’ existing range and assessments made over time. We have now clarified this in the Abstract:

*The current study is the first to assess the impact on beer sales of removing the largest draught serving size (one imperial pint) from the options available in licensed premises under real-world conditions. (page 2, lines 56-57)*

**Introduction
2. The last couple of sentences could be a little clearer, you say that a schooner is a pint, but also that Western Australia is transitioning from pints to schooners. Think this is down to regional differences in terminology but rewording could make this clearer.**

We have updated the manuscript as follows to hopefully clarify:

*In South Australia this size is called ‘a pint’ and is therefore the biggest most popular size on offer. (page 5, lines 205-206)*

**3. It would be helpful to mention compensation in the introduction. If people have smaller drinks in the on-trade are they more likely to go home and have more drinks because they'd only had a small one? The Kersbergen study gets at this a bit.**

As our study did not assess possible compensation effects in home-settings, we think that discussion of this possibility is better suited to the Discussion section. We have added this possibility to the manuscript in response to the reviewer’s last comment:

*Finally, it is unknown whether people compensated for their reduced beer consumption by drinking more alcohol at home, although this has not been shown to be the case in previous research, in which, like in the current study, pints were replaced by 2/3 pints during one drinking occasion in a semi-naturalistic setting [28].(page 15, lines 658-623)*

**4. Theres also a theory about norms and serving size, which outlines that there is a range of serving sizes which are accepted as being one serving,** [**https://eur03.safelinks.protection.outlook.com/?url=https%3A%2F%2Fwww.ncbi.nlm.nih.gov%2Fpmc%2Farticles%2FPMC6333281%2F%23%3A~%3Atext%3DThe%2520first%2520proposition%2520of%2520the%2C%27not%2520normal%27%2520in%2520size&data=05%7C02%7Ctm388%40medschl.cam.ac.uk%7Cc6a6b3cd99244628a75908dc59589d6c%7C49a50445bdfa4b79ade3547b4f3986e9%7C1%7C0%7C638483484346194604%7CUnknown%7CTWFpbGZsb3d8eyJWIjoiMC4wLjAwMDAiLCJQIjoiV2luMzIiLCJBTiI6Ik1haWwiLCJXVCI6Mn0%3D%7C0%7C%7C%7C&sdata=qvoRFlSFdWII0amF3hhvmfZPTtMFYwfoDDX8IhN3q0Y%3D&reserved=0**](https://www.ncbi.nlm.nih.gov/pmc/articles/PMC6333281/#:~:text=The%20first%20proposition%20of%20the,'not%20normal'%20in%20size) **whereby compensation is less likely, which I believe Dr Kersbergen has applied to alcohol consumption too. (I am not Dr Kersbergen but a big fan of her research).**

As mentioned in our response to the comment above, given that the study didn’t attempt to assess compensation effects in homes, we believe this discussion is also better suited to the Discussion. We have now referenced the study the reviewer helpfully mentioned and also added the following to the manuscript:

*Given that serving sizes perceived as normal do not lead to compensation effects, in contrast to when sizes are perceived as small [64], future research should also assess the possible mediating effect of social norms about serving sizes. This could mean on the potential for people to compensate for reduced consumption in licensed premises by drinking more at home; although previous research [28]. (page 16, 710-715).*

**5. What was the reasoning for only reducing one beverage type? Might it be more effective to remove all the largest servings e.g. wine, spirits.**

We agree with the reviewer that removing larger sizes for all types of drinks might be necessary for tackling alcohol consumption in populations. We have already shown in a previous study that removing the largest serving size of wine by the glass is effective in reducing wine consumption https://journals.plos.org/plosmedicine/article?id=10.1371/journal.pmed.1004313. Before attempting to target drinks across the board, we wanted to assess whether similar effects could be obtained when targeting another popular drink i.e. beer and a well-established serving size such as the imperial pint. The reason for focusing on one drink type at a time is also pragmatic. It is challenging to run these kinds of studies. For premises it is logistically difficult to implement the required changes (change menus and signs, train staff etc) and targeting more than one drink type would make participating even harder. Convincing premises to removing serving sizes is also challenging (hence the low study response rate) due to the expected loss in sales. This would become even more challenging if attempting to remove serving sizes for multiple drinks.

**Methods
6. Did you include draft cider too? You refer to draft beer throughout but seems it would make sense to include cider too? After reading the measures I think cider was included in your primary outcome, this could be clearer throughout including the title - e.g. beer and cider sales.**

Yes we did include cider, as well as beer and lager. As already mentioned in the measures section, our primary outcome was “Daily volume (in millilitres (ml)) of all beer, lager and cider (draught as well as bottled and canned), extracted from electronic records of sales.” We have now included mention of cider and lager in the aim (in the Abstract and Introduction):

*The current study is the first to assess the impact on beer, lager and cider sales (page 2, line 55).*

*The aim of the current study is to assess the impact on the volume of beer, lager and cider sold of removing the largest serving size of draught beer, lager and cider (one imperial pint, 568ml), so that the largest serving size available is two-thirds of a pint. We hypothesise that this intervention reduces the volume of beer, lager and cider sold. (page 5, lines 212-215).*

We have also added information in the Abstract and Methods to explain that beer, lager and cider will be collectively referred to as “beer” for ease:

Abstract: *The current study is the first to assess the impact on beer, lager and cider sales (hereafter, for ease, referred to just as “beer sales”) (page 2, lines 55-56)*

Methods: *draught beer, cider and lager -- hereinafter referred to collectively as “beer” for ease – (page 6, line 258)*

**7. Why didn't you include any PPI input? I guess it's a journal requirement but feels strange to include a section if you don't actually have any PPI input, explaining why you didn't might be helpful. I also think getting some PPI input into these findings and what they actually mean in terms of practice and implementation could be a really interesting addition if you did want to incorporate some PPI.**

The Patient and Public Involvement section was included as it’s the requirement of some journals to do so. We have now removed it to avoid confusion.

**Results
8. It is unclear to me why in your sensitivity analysis with intervention as a unique predictor that your mean difference is less in ml but the proportional decrease is much greater? I can't work out why the total ml would change.**

The model used in sensitivity analysis 1 contains a single covariate, the intervention, while the model used in the main analysis controls for several covariates (as pre-defined in the protocol). Consequently, the intercepts of both models are different in meaning and estimate:

- in the main analysis, the intercept equals 30.617 and corresponds to the average daily volume of beer sold (on the cube root scale) in an average pub with average revenue level, on an average day of the week of the baseline period with average temperature and without special event. As the corresponding intervention effect equals –1.014, the relative decrease can be defined as (1- (30.617- 1.014)^3 / 30.617^3)*100 = 9.6%
- in sensitivity analysis 1, the intercept equals 22.298 and corresponds to the average daily volume of beer sold (on the cube root scale) in an average pub during the baseline period, irrespective of all other predictors (temperature, revenue, special events, day of the week). As the corresponding intervention effect equals –1.069, the relative decrease can be defined as (1- (22.298- 1.069)^3 / 22.298^3)*100 = 13.7%,

where the differences with the results available in the text are due to the rounding used here. Relative to the intercept, the intervention effect is smaller when controlling for further predictors (though non-significantly as the confidence intervals overlap), suggesting that a part of the intervention effect noted in the simple model is due to predictors considered in the main analysis.

The purpose of our sensitivity analyses is to explore how robust our conclusions are to different model assumptions, in this case a different modelling of the primary endpoint. We can note that our conclusions remain the same: the intervention has a significant effect on consumption.

**Discussion
9. I really enjoyed your discussion, all of the things I was thinking about whilst reading the paper are unpicked and explained really nicely. (I had to go back through and delete a lot of my comments as you had done such a good job of unpicking and explaining it all!).
One additional limitation around compensation, you don't know what people drank after, did they compensate at home because they'd only had a small one?**

We appreciate the positive feedback the reviewer has given us. We have now added the possibility of compensation by drinking more at home to the Discussion:

*Finally, it us unknown whether people compensated for their reduced beer consumption by drinking more alcohol at home, although this has not been shown to be the case in previous research, in which, like in the current study, pints were replaced by 2/3 during one drinking occasion in a semi-naturalistic setting [28].(page 15, lines 658-623)*
