# Supplementary material for: Impact on beer sales of removing the pint serving size: An A-B-A reversal trial in pubs, bars, and restaurants in England
Source: PLoS Med. 2024 Sep 17;21(9):e1004442. doi: 10.1371/journal.pmed.1004442 (PMC11407663; doi:10.1371/journal.pmed.1004442)
Supplement: Attachment — Submitted filename: Pints study_response to reviewers 2 FINAL.docx [file pmed.1004442.s006.docx]

Dear Editor,

In response to your email dated 5^th^ July 2024 regarding our manuscript entitled “Impact on beer sales of removing the pint serving size: an A-B-A reversal trial in pubs, bars and restaurants in England”, please find below our responses to the Editor (3 points).

We are grateful for the opportunity to address these many helpful points to generate a stronger manuscript.

Yours Faithfully,

The authors

**Requests from the editors:

Thank you for your detailed response to the editors' and reviewers' comments. I have discussed the paper with my colleagues and the academic editor, and it has also been seen again by the original reviewers. The changes made to the paper were welcomed by the reviewers.

I only have a few minor request for you at this stage:

1) Statistical reporting:
We suggest reporting statistical information in the following format: ‘x’; (95% CI [‘y’,’ z’] p value). For p values, please report as p<0.001 and where higher as 'p=0.002'. Please add the statistical method used to your method section. We also invite you to report p values to consistently to the third decimal digit - thousandths. For example, see abstract : "After adjusting for pre-specified covariates, the intervention resulted in a mean daily change of -2769ml (95% CI -
4188 to -1578, p<0.00001) or -9.7% (95% CI -13.5% to -6.1%) in beer sold. The daily volume of wine sold increased during the intervention period by 232ml (95% CI 13 to 487, p=0.035) or 7.2% (95% CI 0.4% to 14.5%). Daily revenues decreased by 5.0% (95% CI -9.6% to -0.3%, p=0.038). " Please check and amend throughout.**

We have amended the manuscript throughout according to the Editor’s suggestions. The statistical method used for the primary analysis was already reported in the Methods section as “a heteroscedastic linear mixed model” (page 9, line 383). We have added more information regarding the Sensitively analyses 1-3 to be more explicit in the method used (heteroscedastic linear mixed model) (page 10, lines 408, 413, 419).
 **2) CONSORT checklist. Thank you for supplying the CONSORT checklist (S3). Please remove the page numbers from this list, and use section headers and paragraph numbers, please remove the page numbers.**

We have now removed page number from the CONSORT checklist. **3) please double check that reference #29 is updated**

We thank the Editor for the observation that this reference was incomplete. We have not updated it. **------------------------------------------------------------

Comments from Reviewers:

Reviewer #1: Thanks for the revised manuscript and responses to my original review. The updated manuscript resolves my original queries - the more detailed figures in the appendix are a helpful addition


Reviewer #2: Thankyou, I am happy with the revisions and response to my comments.


Reviewer #3: The authors have responded to all my comments and I would recommend that this paper is now published.**

We thank all the reviewers for the positive feedback.
